# Proximal Disruption of Brain Energy Supply Raises Systemic Blood Glucose: A Systematic Review

**DOI:** 10.3389/fnins.2021.685031

**Published:** 2021-06-24

**Authors:** Marie Sprengell, Britta Kubera, Achim Peters

**Affiliations:** Medical Clinic 1, Center of Brain, Behavior and Metabolism (CBBM), University of Lübeck, Lübeck, Germany

**Keywords:** brain energy metabolism, cerebral artery occlusion, blood glucose, cerebral insulin suppression, selfish-brain theory, systematic review

## Abstract

This work joins a series that methodically tests the predictions of the Selfish-Brain theory. The theory postulates a vital ability of the mammalian brain, namely to give priority to its own energy metabolism. The brain behaves “selfishly” in this respect. For the cerebral artery occlusion studied here, the theory predicts an increase in blood glucose concentration, what becomes the hypothesis to be tested. We conducted a systematic review of cerebral-artery-occlusion papers to test whether or not the included studies could confirm this hypothesis. We identified 239 records, screened 231 works by title or abstract, and analyzed 89 by full text. According to strict selection criteria (set out in our PROSPERO preregistration, complying with PRISMA guidelines), 7 papers provided enough information to decide on the hypothesis. Our hypothesis could be fully confirmed for the 3 to 24 h after the onset of a transient 2 h or permanent occlusion. As for the mechanism, the theory predicts that the energy-deprived brain suppresses insulin secretion via the sympathoadrenal system, thereby preventing insulin-mediated glucose uptake into muscle and fat and, as a result, enhancing insulin-independent glucose uptake via the blood-brain barrier. Evidence from our included studies actually demonstrated cerebral insulin suppression. In all, the current work confirms the second major prediction of the Selfish-Brain theory that relates to a proximal bottleneck of the cerebral supply chain, cerebral artery occlusion. Its first major prediction relates to a distal supply bottleneck, caloric restriction, and is fulfilled as shown by our previous work, whereas the prediction of the long held gluco-lipostatic theory, which sees the brain as only passively supplied, is violated (Sprengell et al., 2021). The crucial point was that caloric restriction elicits smaller changes in mass (energy) in the brain than in the body. Taken together, the evidence from the current and previous work clearly shows that the most accurate predictions are possible with a theory that views the brain as an independently self-regulating energy compartment occupying a primary position in energy metabolism.

## Introduction

**Figure 1:**
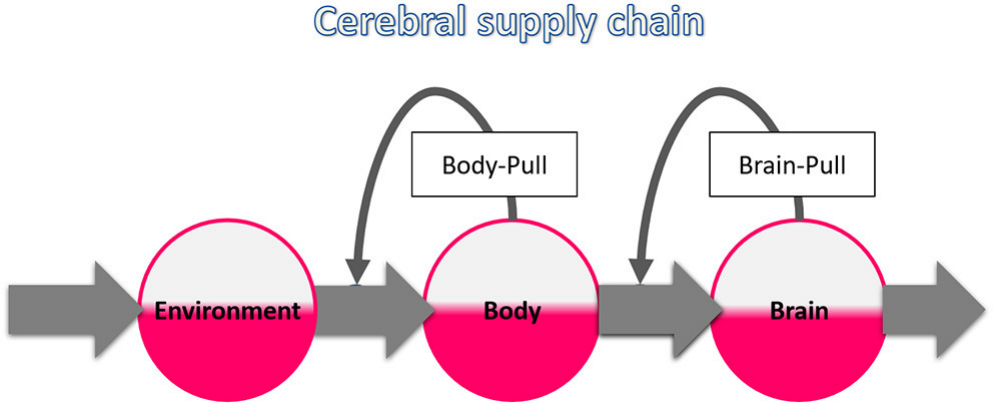
The cerebral supply chain. Energy is transferred from the environment through the body to the brain, the end consumer of the supply chain. The characteristic of supply chains is the procurement on demand, also called pull principle. When the brain needs energy, the brain-pull demands it from the body. When the body needs energy, the body-pull demands it from the environment.

This work is based on the concept of the “cerebral supply chain” where the brain is the end consumer (Peters and Langemann, 2009) (Figure 1). The key principle of supply chains is on-demand procurement, which makes the delivery process more economical and less susceptible to disturbances (Slack et al., 2004). We performed a systematic review on the most critical disturbance of the cerebral supply chain: a proximal supply bottleneck caused by cerebral arterial occlusion. It is known that disruption of cerebral fuel supply results in an intraneuronal adenosine triphosphate (ATP) depletion that ultimately leads to ischemic stroke (Wagner et al., 1992). It is also known that patients with acute ischemic stroke often present with hyperglycemia (Bravata et al., 2003), but it remains unclear to what extent this is due to the stroke or undiagnosed diabetes mellitus. What exactly a proximal bottleneck does to the more distal parts of the cerebral supply chain has not yet been studied systematically.

**Figure 2:**
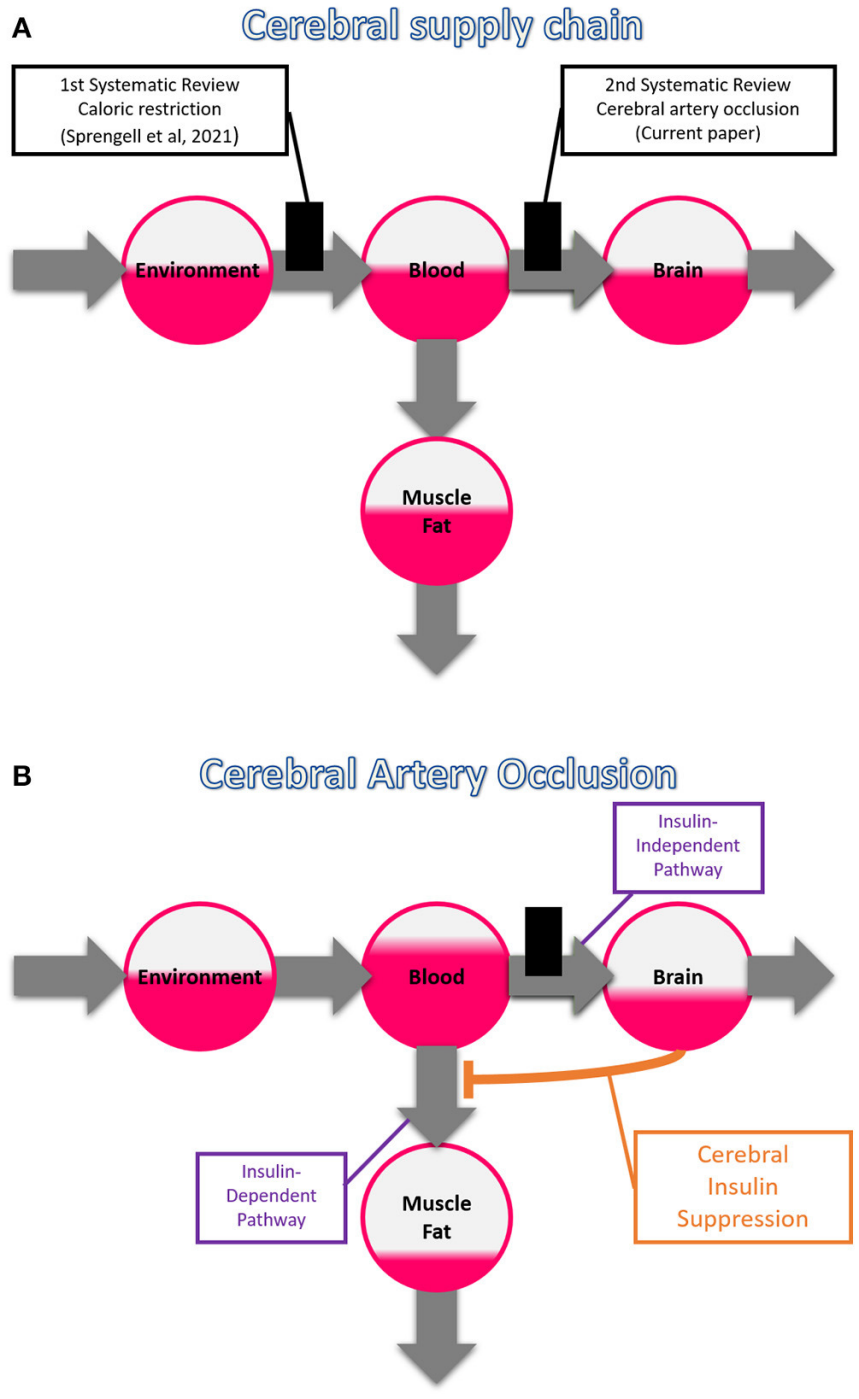
The branched cerebral supply chain. The branching originates from the blood, transmitting energy either to the brain or to muscle or fat tissue. **(A)** Systematic reviews on two different bottlenecks in the cerebral supply chain. The first one deals with a distal bottleneck, i.e. caloric restriction, and is already published (Sprengell et al., 2021); the second one, the current work, deals with a proximal bottleneck, i.e. cerebral artery occlusion. **(B)** Cerebral insulin suppression induced by cerebral artery occlusion. Intraneuronal ATP depletion activates the sympathetic nervous system and the hypothalamic-pituitary-adrenal axis, which both rigorously suppress pancreatic ß-cell secretion, and in so doing block insulin-dependent glucose uptake in muscle and fat tissue. The portion of glucose that is prevented from entering muscle and fat tissue accumulates in the blood, facilitating transfer across the blood-brain barrier via the insulin-independent pathway, allowing partial compensation when brain supply is compromised.

This is our second systematic review on the cerebral supply chain; our first examined the effect of a more distal bottleneck in the supply chain, i.e. caloric restriction (Figure 2A). We found that with caloric restriction, the mass (energy) changes in the brain were particularly small (Sprengell et al., 2021). Thus, peripheral energetic intervention has little effect on the central nervous system. In the current systematic review, we examine the reverse case, namely, what effect central energetic interventions have on peripheral energy metabolism. More precisely, we examined what effect the interruption of the brain's energy supply has on the systemic blood glucose concentration. We hypothesize that disruption of brain arterial supply causes a marked rise in blood glucose concentration.

Our hypothesis is a prediction of the Selfish-Brain theory (Peters et al., 2004). This theory deals with the energy allocation within the mammalian organism. It postulates that in case of energy shortage there is a vital ability of the brain, namely to give priority to its own energy metabolism. Rival positions include the long-held gluco-lipostatic theory (Kennedy, 1953; Mayer, 1953) and its modern variants (Chaput and Tremblay, 2009; Schwartz et al., 2017), which all view the brain as only passively supplied. Our first systematic review showed that caloric restriction causes only minor mass (energy) changes in the brain [of the order of −4%] as opposed to major changes in the body [of about −30%] (Sprengell et al., 2021). This finding completely fulfilled the predictions of the Selfish-Brain theory, while those of the gluco-lipostatic theory and its variants were violated. The most accurate predictions are therefore possible with a theory that views the brain as an independently self-regulating energy compartment that occupies a primary position in a hierarchically organized metabolism. Our second systematic review, the current one, aims to examine evidence of this vital ability of the brain. That is, if the hypothesis formulated here can be confirmed by our systematic literature search, an important prediction of the Selfish-Brain theory is fulfilled.

To illustrate how our hypothesis can be deduced from theory, we refer to the cerebral supply chain. It is a mathematical representation of the Selfish-Brain theory (Peters and Langemann, 2009). Logistic supply chains have played an important role in the economy for 80 years, e.g. in automobile production. The mathematical principles of these supply chains could be directly applied to energy metabolism. A general rule for all supply chains is that the flow of goods or energy is composed of two components, one determined by the supplier (push component) and the other by the subsequent recipient (pull component) (Slack et al., 2004). In the cerebral supply chain, energy is transferred from the environment, through the blood, to the end consumer – the brain. As this supply chain is branched, a portion of the energy is transferred from the blood to the muscle and fat tissue. In addition to the antegrade flow of energy toward the brain as the end consumer, there is also a retrograde flow of information consisting of the pull commands.

Brain-pull refers to the brain's ability to procure itself with energy on demand (Figure 1). The energy content of the brain (intraneuronal ATP) determines how much energy is demanded from the body. In contrast, the body-pull demands energy from the environment for the body. Both the energy content of the blood (glucose) and that of the body stores (triglycerides) determine how much energy is pulled from the environment. To date, several redundant brain-pull mechanisms have been identified (Peters and McEwen, 2015). These are neuroendocrine in nature and provide the brain with additional energy sources as needed.

Among brain-pull mechanisms, “cerebral insulin suppression” (CIS) is one of the most important (Woods and Porte, 1974; Hitze et al., 2010). Energy sensors in brain regions such as the amygdala (Zhou et al., 2010) and VMH (Spanswick et al., 1997; Routh et al., 2014; Toda et al., 2016) detect even the slightest drop in neuronal ATP and set brain-pull mechanisms in action. VMH activation lowers insulin concentrations and increases blood glucose concentrations (Meek et al., 2016; Stanley et al., 2016). In detail, the VMH activates the sympathoadrenal system, which strongly suppresses insulin secretion from pancreatic ß-cells (Ahren, 2000). Insulin-dependent glucose uptake (GLUT4) in muscle and fat tissue is suppressed, while the remaining circulating glucose is almost completely available to insulin-independent glucose transport (GLUT1) across the blood-brain barrier. For the brain needs virtually no insulin to take up glucose (Hom et al., 1984; Hasselbalch et al., 1999; Seaquist et al., 2001). In summary, GLUT1 glucose uptake safeguards basal energy supply of vital organs, like brain and immune cells (Deng et al., 2014), while GLUT4 allows the storage of surplus energy in muscle and fat cells (Shepherd and Kahn, 1999). Thus, CIS allocates more energy to the brain when needed.

In the case of cerebral artery occlusion, our theory-based prediction is as follows (Figure 2B). Occlusion of a cerebral artery restricts the energy flow to the brain, which in the supply chain model corresponds to a reduced blood-push component. Reduced supply (i.e. decreased blood-push) leads to cerebral ATP depletion, resulting in enhanced CIS (i.e. increased brain-pull), which prevents glucose uptake in muscle and fat tissue, and eventually causes glucose accumulation in the blood. While a portion of the circulating glucose cannot enter muscle or fat tissue, this is instead made available to the energy-depleted brain. In this way, the cerebral supply chain model generates a prediction that becomes the hypothesis to be tested here: *Cerebral artery occlusion increases glucose concentration in the blood*.

To this end, we performed a systematic review to examine whether or not the experimental studies found on cerebral artery occlusion can actually confirm this hypothesis.

## Materials and Methods

The protocol for this systematic review was pre-registered on PROSPERO on 30th of January 2020, and updated versions were published on 28th of September 2020 and 14th of December 2020 (International prospective register of systematic reviews; CRD42020156816). We complied with the PRISMA (Preferred Reporting Items for Systematic Reviews and Meta-analyses) guidelines for systematic reviews of interventions (Moher et al., 2009). Furthermore, the Cochrane Handbook for systematic reviews of interventions was used (Higgins and Thomas, 2019).

### Search Strategies

We conducted a systematic search of the literature to identify studies in mammals that focused on how the experimental cerebral artery occlusion affects blood glucose concentration. One reviewer developed the search strategies, which were then discussed with two other reviewers. The databases of MEDLINE and BIOSIS Previews were searched from their inception to 20th December 2020, using a combination of key words and in case of the first database MeSH terms. The full MEDLINE and BIOSIS search strategies are provided in the Supplementary Material. Briefly, the search strategies included terms relating to the intervention (cerebral ischemia), to the outcome (blood glucose concentration) and to the methodical approach (experimental study), combined by the Boolean operator AND. Synonyms for terms were combined with the operator OR.

### Study Selection

We used the following criteria to include or exclude articles for our systematic review. Only original full research papers published in English or German were included. We included studies that examined mammals of any species or sex. We included only interventional studies that were standardized laboratory experiments or clinical trials and that examined two groups, an interventional group undergoing cerebral artery occlusion and a non-exposed, sham-operated control group. Since we had included clinical trials in our first systematic review (Sprengell et al., 2021), we did the same here for the sake of consistency, but of course did not expect to identify clinical trials, since cerebral artery occlusion in humans is not ethically defensible. We only included studies that measured blood glucose concentration. We did not include studies that measured outcomes in the intervention and control groups at different time points. We did not include studies, that occluded the vertebral arteries, as cerebral control centers of energy metabolism could have been affected. We did not include studies, in which clots were injected in the aorta, because of unpredictable consequences for other parts of the body. We did not include studies with combined interventions such as cerebral artery occlusion and systemic hypotension. We did not include studies in which the individuals had diseases or were on medication that had been shown to affect energy metabolism; for more details see Sprengell et al. (2021). We did not include trials in pregnant individuals or fetuses, nor in ovariectomized or genetically modified mammals with altered energy metabolism.

**Figure 3:**
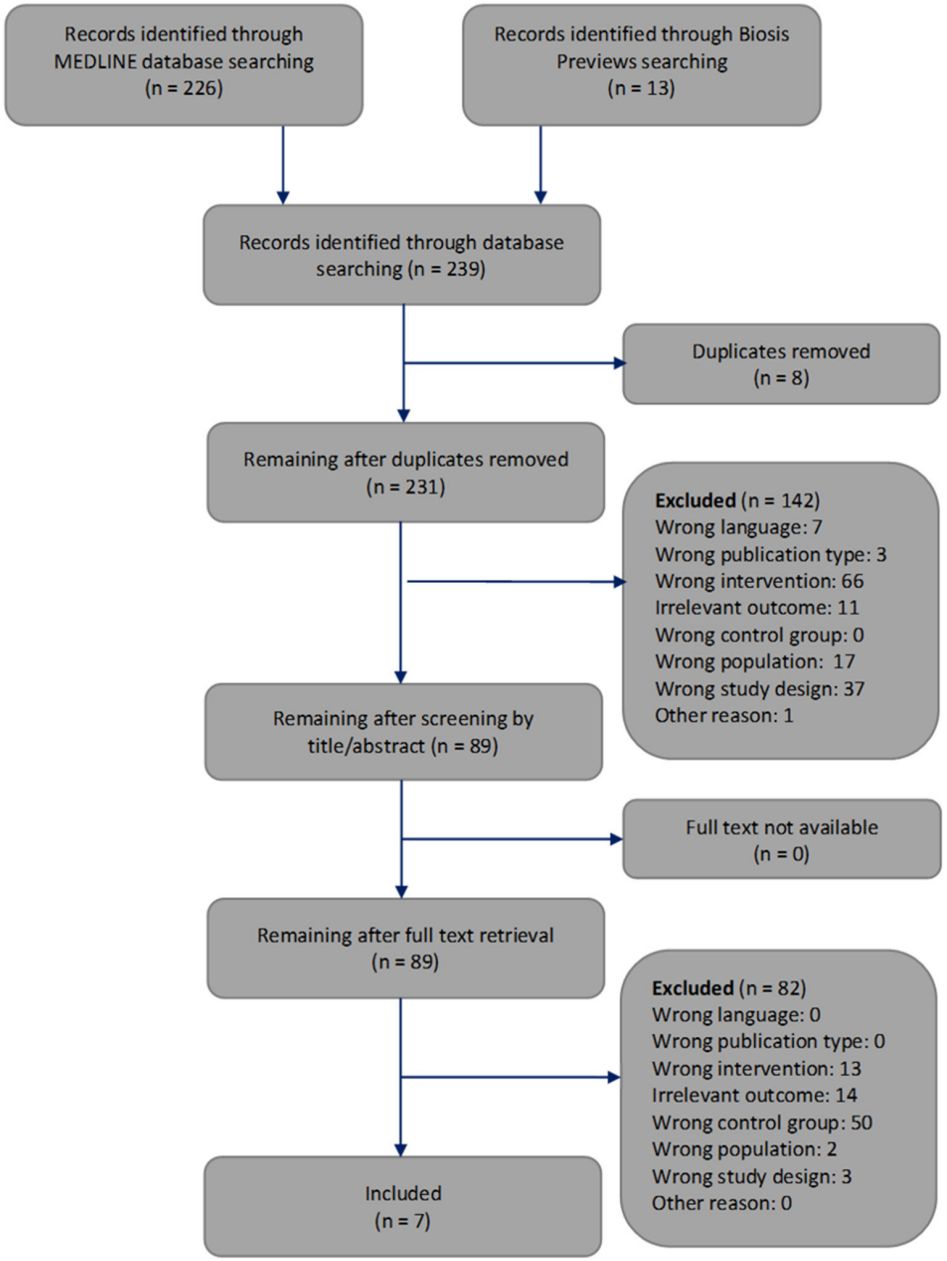
Flowchart through different phases of the systematic review modified according to Moher's publication (Moher et al., 2009).

The selection of the articles was performed in two steps. We have indicated the reasons for exclusion at each step (Figure 3). First, one reviewer screened the article titles or abstracts against the inclusion and exclusion criteria. This first step of article selection was checked by another reviewer. When a discrepancy occurred regarding the inclusion or exclusion of an article, the two reviewers discussed it until agreement was reached. Otherwise, disagreements were resolved by consulting the third reviewer. Second, two reviewers independently selected the remaining articles by analyzing the full text. Again, disagreements regarding the inclusion or exclusion of an article were resolved by discussion among each other or, if necessary, by consultation with the third reviewer.

### Data Extraction

Data from all of the 7 included studies were extracted by one reviewer, independently checked by two other reviewers, and tabulated alphabetically. We recorded the population, sample size, kind of intervention and duration, study design, statistical test applied, baseline blood glucose concentrations as well as blood glucose concentrations after onset of cerebral artery occlusion and secondary outcomes.

### Risk of Bias Assessment

To assess the risk of bias of non-human studies the SYRCLE‘s tool was used (Hooijmans et al., 2014). One reviewer assessed the risk of bias of the 7 included studies. The results were independently checked by two other reviewers. All differences were clarified by discussion.

### Hypothesis Decision

Based on the respective statistical test, we decided whether the hypothesis could be confirmed (p < 0.05) or not (p ≥ 0.05).

## Results

The systematic search of the literature generated 239 articles, which were processed as summarized in Figure 3. Two hundred and thirty one works were screened by title or abstract, and 89 articles were analyzed by full text. We identified seven studies that met all inclusion criteria and focused on how cerebral artery occlusion affects blood glucose concentrations (Harada et al., 2009; Yamazaki et al., 2012, 2014; Wang et al., 2013, 2014; Li et al., 2016; Boujon et al., 2019).

### Data Extraction

Table 1 provides details of the 7 included studies. Four studies investigated mice (Harada et al., 2009; Yamazaki et al., 2012, 2014; Boujon et al., 2019) and 3 studies investigated rats (Wang et al., 2013, 2014; Li et al., 2016). The sample sizes varied between 9 and 53.

**Table 1:** Characteristics and results of included studies.

**References**	**Popula-tion**	**Sample size**	**Intervention**	**Design**	**Statistical test**	**Blood glucose [mg/dL]**				**Secondary outcomes of energy metabolism**
						**Baseline**	**Early Phase <3h after occlusion onset**	**Intermediate Ph. ≥ 3 and ≤ 24h after occl. onset**	**Late Phase > 24h after occl. onset**	
Boujon et al. (2019)	129S6/ SvEv mice, ~ 10 weeks old, male	Exp: 5^a^ Con: 4	**30-min transient** occlusion of left middle cerebral artery and ant. choroidal arteries by inserting nylon monofilament	BG was measured at baseline, on day 3, and on day 7	Two-way ANOVA followed by Turkey's multiple comp. test. Mean ±S.E.M.	Exp: 110 ±^sup>b^ Con: 120 ±4^ns,^^b^			**Day 3** Exp: 76 ± 4^b^ Con: 84 ± 6^ns,^^b^ **Day 7** Exp: 117 ± 14^b^ Con: 128 ± 9^ns,^^b^	
Harada et al. (2009)	ddY mice, 5 weeks old, male	Exp: 8-17^c^ Con: 9-16	**2-h transient** occlusion of the left middle cerebral artery through insertion of nylon monofilament; ligation of the left common carotid artery and external carotid artery	BG was measured at baseline and afterwards at 1h, 3h, 6h, 12h, on day 1, 3 and 5. Increment of BG was calculated.^d^	One-way ANOVA followed by paired student's *t*-test. Data are shown as mean ± S.E.M.^e^	Exp: 0±s^ns,^^b,d,e^ Con: 0±s^b,d,e^	**1h** Exp: 28±s^b,d,e^ Con: 38 ± 5 ^ns,^^b,d^ (increment)	**3h** Exp: 20±s^b,d,e^ Con: 25±s ^ns,^^b,d,e^ **6h** Exp: 9±s^b,d,e^ Con: 16±s ^ns,^^b,d,e^ **12h** Exp: 63 ± 5^b,d^ Con: 14±s^**^^b,d,e^ **D1** Exp: 74 ± 16^b,d^ Con: 22±s^**^^b,d,e^ (increment)	**Day 3** Exp: 22^b,d,f^ Con: 22^ns,^^b,d,f^ **Day 5** Exp: −13 ± 14^b,d^ Con: 16±s^ns,^^b,d,e^ (increment)	Insulin, insulin after glucose load
Li et al. (2016)	Sprague-Dawley rats, male	Exp: 23^g^ Con: 17	**2-h transient** occlusion of the left middle cerebral artery with nylon suture	BG was measured before, during, and after 2h occlusion	One-way ANOVA	Data not shown	Data not shown. Q: “no significant changes of …. glucose levels… between different groups”; test statistics not shown.			Body weight (data not shown)
Wang et al. (2013)	Sprague- Dawley rats, adult, male	Exp: 6 Con: 6	**Permanent** occlusion through clamping of the 2 common carotid arteries and right middle cerebral artery	BG was measured hourly over 24h	Student‘s *t*-test. Data are shown in the original paper as mean ± S.D. They were converted by the present reviewers to S.E.M.^h^	Exp: 83^b^^,^^f^ Con: 83^b^^,^^f^	**1h** Exp: 100 ± 2^b^^,^^h^ Con: 82 ± 2^**^^b^^,^^h^ **2h** Exp: 103 ± 2 ^b^^,^^h^ Con: 77 ± 1^**^^b^^,^^h^	**3h** Exp: 93 ± 3 ^b^^,^^h^ Con: 86 ± 2^*^^b^^,^^h^ **4h** Exp: 97 ± 4 ^b^^,^^h^ Con: 83 ± 2^**^^b^^,^^h^ **5h** Exp: 97 ± 3 ^b^^,^^h^ Con: 86 ± 2^*^^b^^,^^h^ **6h** Exp: 98 ± 3^b^^,^^h^ Con: 78 ± 2^**^^b^^,^^h^ **7h** Exp: 102 ± 3^b^^,^^h^ Con: 79 ± 2^**^^b^^,^^h^ **8h** Exp: 104 ± 4^b^^,^^h^ Con: 77 ± 1^**^^b^^,^^h^ **9h** Exp:106 ± 5^b^^,^^h^ Con: 79 ± 4^**^^b^^,^^h^ **10h** Exp: 108 ± 4^b^^,^^h^ Con: 78 ± 2^**^^b^^,^^h^ **11h** Exp: 104 ± 5^b^^,^^h^ Con: 81 ± 2^**^^b^^,^^h^ **12h** Exp: 116 ± 5^b^^,^^h^ Con: 82 ± 2^**^^b^^,^^h^ **24h** Exp:98 ± 4^b^^,^^h^ Con: 73 ± 2^**^^b^^,^^h^		Cortisol, glucagon, fasting insulin
Wang et al. (2014)	Sprague- Dawley rats, adult, male	Exp: 8 Con: 8	**Permanent** occlusion by clamping the 2 common carotid arteries and right middle cerebral artery	BG was measured at baseline and on day 1	Student's unpaired *t*-test. Data are shown as mean ± S.E.M.	Exp: 81.1 ± 5.4^ns,^^i^ Con: 79.3 ± 7.2^i^		**Day 1** Exp: 106.3 ± 5.4^i^ Con: 81.1 ± 7.2^*^^i^		Body weight, fasting insulin, epinephrine, norepi-nephrine
Yamazaki et al. (2012)	ddY mice, 5 weeks old, male	Exp_1_: 17 Con_1_: 12 Exp_2_: 16 Con_2_: 8	**2-h transient** occlusion of the left middle cerebral artery through insertion of nylon mono-filament	BG was measured at baseline and on day 1. Increment of BG was calculated^d^	One-way ANOVA followed by Scheffe's test. Mean ±S.E.M.	Not shown		**Day 1** Exp_1_: 65 ± 5^b,d^ Con_1_: 14 ± 7^**^^b,d^ (increment) **Day 1** Exp_2_: 60 ± 3^b,d^ Con_2_: 28 ± 5^**^^b,d^ (increment)		
Yamazaki et al. (2014)	ddY mice, 5 weeks old, male	Exp_1_: 7 Con_1_: 9 Exp_2_: 6 Con_2_: 7	**2-h transient** occlusion of the left middle cerebral artery through insertion of nylon mono-filament	BG was measured at baseline and on day 1. Increment of BG was calculated^d^	One-way ANOVA followed by the Scheffe's *post-hoc* test. Mean ±S.E.M.	Not shown		**Day 1** Exp_1_: 44 ± 6^b,d^ Con_1_: 13 ± 5^*^^b,d^ (increment) **Day 1** Exp_2_.: 43 ± 3^b,d^ Con_2_: 13 ± 5^**^^b,d^ (increment)		

*Only information from study arms meeting our inclusion criteria is presented. The sample sizes refer to the number of animals that survived the intervention and the observation period and whose outcomes were measured. Secondary outcomes relevant to our research question are shown in the last column. For time data, onset of ischemia is considered timepoint 0. Data are presented as means ± S.E.M. Abbreviations: Exp: experimental group; Con, Control group; BG, blood glucose; ns, not significant; Q.:Quote; S.D.: Standard deviation; S.E.M.: Standard error of the mean*

* *p < 0.05*,

** *p < 0.01*.

a *One mouse out of six of the interventional group died*.

b *Value taken from graph. Some of the values could only be estimated*.

c *For the time course experiments, the authors used independent 8–17 mice in every indicated period; that is, they euthanized different numbers of animals at 6 h after the occlusion onset, 12 h, 1 day, 3 day, or 5 day and obtained the respective measurements*.

d *Increment of blood glucose was determined by authors using the following formula: increment of BG = BG after occlusion – BG before occlusion*.

e *Certain data points within the graphic were displayed so large that they concealed the S.E.M. markers. We estimate the radius of these graphically displayed points to be 5. The variable s stands accordingly for a S.E.M. of ≤ 5mg/dL*.

f *Estimation of variances from the figures is not possible if the graphically represented data points of the intervention and control groups overlap*.

g *Four out of 27 rats of the interventional group died*.

h *The standard deviation was presented in the original paper. It was converted by the present reviewers to the standard error of the mean using the following formula: $S.E.M.\;=\;\frac{Standard\;Deviation}{\sqrt{Sample\;Size}}$. The S.E.M. values have been rounded to whole numbers and are presented in the above table*.

i *Glucose concentration is expressed in the unit mM in the original paper. The present reviewers have multiplied the concentration value by the conversion factor 18.02*.

All included studies provided details on how cerebral artery occlusion was implemented. In 2 studies, the left middle cerebral artery was occluded by insertion of a nylon monofilament (Yamazaki et al., 2012, 2014). Harada and colleagues also occluded the left middle cerebral artery and additionally ligated the left common carotid artery and external carotid artery (Harada et al., 2009). Two other studies performed occlusion by clamping the two common carotid arteries and right middle cerebral artery (Wang et al., 2013, 2014). In one study, the left middle cerebral artery and anterior choroidal arteries were occluded by insertion of a nylon monofilament (Boujon et al., 2019). In a further study, the middle cerebral artery was occluded with nylon suture (Li et al., 2016). The duration of cerebral artery occlusion ranged from 30 min (Boujon et al., 2019) and 2 h (Harada et al., 2009; Yamazaki et al., 2012, 2014; Li et al., 2016) to permanent (Wang et al., 2013, 2014).

Blood glucose concentrations were measured during three phases, where we distinguished the early phase lasting less than 3 h after occlusion onset, the intermediate phase 3 to 24 h after occlusion onset, and the late phase lasting more than 7 days after reperfusion. Work examining the intermediate phase included, firstly, a paper by Wang and coworkers, which reported hourly measurements over 24 h of permanent ischemia, finding that as early as 1 h after the onset of occlusion, the blood glucose profile was elevated and remained elevated above that of controls (all p < 0.05) (Wang et al., 2013); secondly, another paper by Wang and coworkers, which reported measurements of blood glucose concentrations at baseline and after 24 h of permanent ischemia, finding that 24 h after the onset of occlusion, blood glucose was higher than in controls (p < 0.05) (Wang et al., 2014); thirdly, 2 papers by Yamazaki and coworkers, each reporting in 2 experiments the change in blood glucose concentration 24 h after the onset of a transient 2 h cerebral artery occlusion, finding that blood glucose increased more than in controls (all p < 0.05) (Yamazaki et al., 2012, 2014); and finally the work by Harada and coworkers that examined not only the intermediate phase, but also early and late phase, reporting changes in blood glucose concentration at baseline and 1, 3, 6, 12, and 24 h, 3 and 5 days after the onset of transient 2 h cerebral artery occlusion, finding that the blood glucose increase was more pronounced than in controls at 12 and 24 h (p < 0.01), while no increase could be detected within the first 6 h or on day 3 or 5 (Harada et al., 2009).

Work examining only the late phase was that of Boujon and co-workers, which reported measurements of blood glucose concentration at baseline and on days 3 and 7 after the onset of transient 30 min cerebral artery occlusion, and found that no increase could be detected on either day 3 or 7 (Boujon et al., 2019). Work examining only the early phase was that of Li and coworkers, who could not demonstrate a difference in blood glucose levels between the intervention and sham-operated groups either during the 2 h occlusion or right afterward (Li et al., 2016).

Secondary outcomes relevant to our research question were provided by (Harada et al., 2009) (insulin concentrations and insulin after glucose load), (Wang et al., 2013) (cortisol, glucagon, fasting insulin concentrations) and (Wang et al., 2014) (fasting insulin, blood norepinephrine, blood epinephrine concentrations, and body weight).

### Risk of Bias Assessment

Table 2 provides the risk of bias assessments for all 7 included studies.

**Table 2:** Risk of bias assessment.

**References**	**Random sequence generation**	**Baseline characteristics**	**Addressing of Incomplete outcome data**	**Selective outcome reporting**	**Other sources of bias**
Boujon et al. (2019)	+	+^a^	–^b^	+	?^c^
Harada et al. (2009)	?	+^d^	+	–^e^	+^f^
Li et al. (2016)	+	-^g^	–^h^	–^h^	+^f^
Wang et al. (2013)	+^i^	+^a^	+^j^	+	+^f^
Wang et al. (2014)	+^i^	+^a^	?^k^	+	+^f^
Yamazaki et al. (2012)	?	+^d^	+^j^	+	+^f^
Yamazaki et al. (2014)	?	+^d^	+^j^	+	+^f^

*The SYRCLE‘s tool (Hooijmans et al., 2014) was used and slightly modified to assess the risk of bias of non-human studies. “+” indicates a low risk of bias, “–” indicates a high risk of bias and “?” indicates that not enough information has been provided on this point. For five items of the SRYCLE tool, i.e. allocation concealment, random housing, blinding of personnel, random outcome assessment and blinding of the outcome assessors, not enough information was available for any of the studies examined here, so that all of them were rated “?”. Boujon and coworkers reported, that “experiments were carried out in a blinded fashion”, but it remains unclear which procedures this refers to. The item “Selective outcome reporting” refers to data that is related to our research question*.

a *Similar baseline characteristics in blood glucose concentration*.

b *Attrition bias: 1 out of 6 mice (17%) in the MCAO group died; 1 out of 4 mice (25%) in the sham-operated group was excluded because of poor baseline rotarod performance*.

c *First, the study was supported by Roche; Quote: “Roche did not play a role in the conduct of the experiments reported here, nor in the collection, analysis, or interpretation of the data, nor in the preparation of this manuscript.” Second, the anesthetics chloral hydrate and isoflurane used in all included studies may increase blood glucose. All included papers described that they used the same anesthesia in the sham control group, Boujon and coworkers probably did the same, but did not mention it explicitly*.

d *The increment of blood glucose was presented and analyzed*.

e *Quote “We eliminated mice with brain hemorrhage”; effect on results unclear*.

f *No evidence for unequal housing conditions, conflict of interests or problems of the study design*.

g *Neither baseline blood glucose concentrations nor any other blood glucose concentrations shown*.

h *Attrition bias: 4 out of 27 (15%) mice of the intervention group died and were excluded from the analysis, whereas no rat died in the sham-operated control group. Without specifying any test statistics, Li and coworkers reported that they were unable to detect a statistical difference in blood glucose concentration between rats subjected to cerebral ischemia and sham-operated rats. Thus, it cannot be ruled out that the deceased and surviving rats differed in infarct size and blood glucose profile. In this uncertain situation, one would have liked to see the original blood glucose data*.

i *Random allocation into study groups; randomization of subgroup allocation not explicitly mentioned*.

j *No evidence for dropouts*.

k *Initially there were 8 rats per subgroup, the result table mentions 6–8 rats per subgroup; reason remains unclear*.

### Hypothesis Decision

**Figure 4:**
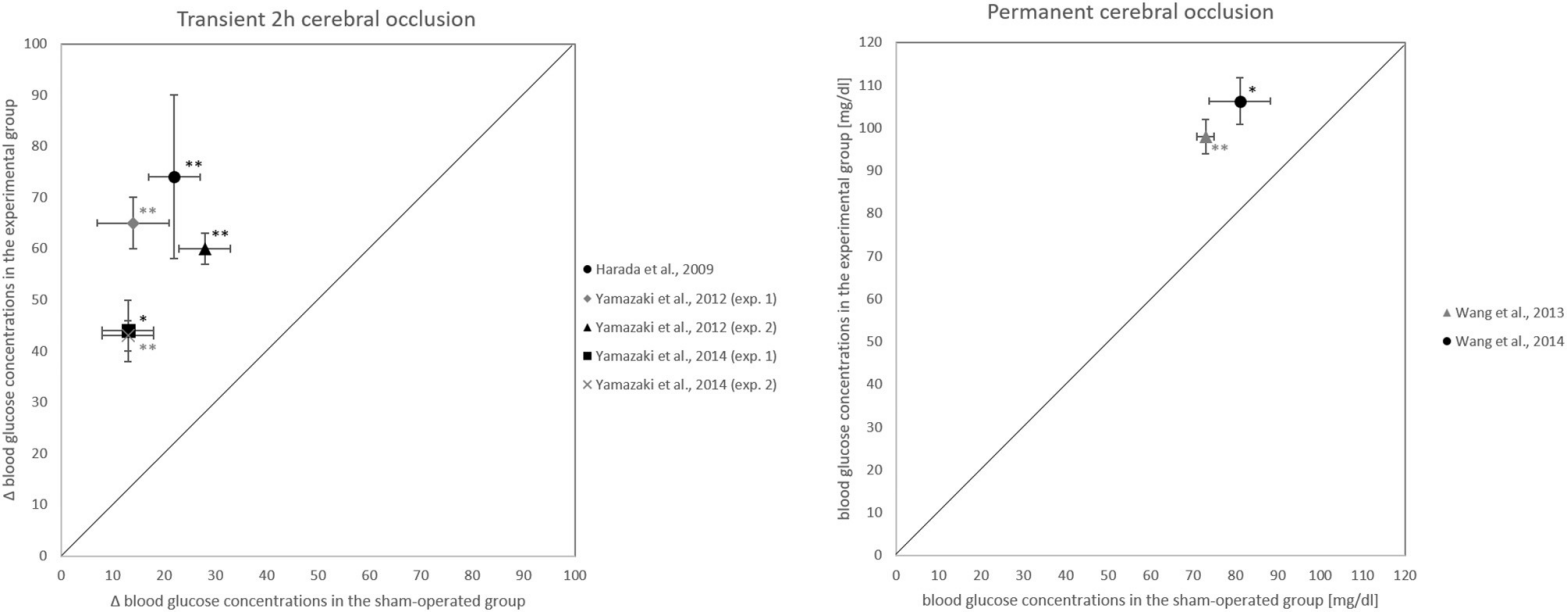
Blood glucose changes 24 h after occlusion onset. **(Left)** Comparison of how experimental and sham-operated groups differ in their blood glucose changes from before to 24 h after the onset of transient 2 h cerebral artery occlusion. **(Right)** Comparison of how experimental and sham-operated groups differ in blood glucose concentrations at 24 h after onset of permanent occlusion. *P < 0.05, **P < 0.01, significant difference in blood glucose concentrations or their changes between experimental and sham-operated groups.

All papers which examined the intermediate phase 3 to 24 h after occlusion onset could confirm our hypothesis (Harada et al., 2009; Yamazaki et al., 2012, 2014; Wang et al., 2013, 2014) (Figure 4). All of these intermediate phase studies showed that blood glucose concentrations were significantly higher or had changed more in the intervention group than in the sham-operated group (Table 3). One work could confirm the hypothesis already for the early phase 1 h after occlusion onset (Wang et al., 2013).

**Table 3:** Hypothesis decision in the different ischemia phases.

**References**	**Early Phase <3 h after onset of occlusion**	**Intermediate Phase≥ 3 and ≤ 24 h after onset of occlusion**	**Late Phase > 24 h after onset of occlusion**
Boujon et al. (2019)			–
Harada et al. (2009)	–	+	–
Li et al. (2016)	–		
Wang et al. (2013)	+	+	
Wang et al. (2014)		+	
Yamazaki et al. (2012)		++^a^	
Yamazaki et al. (2014)		++^a^	

*“+” indicates “hypothesis could be confirmed”, “–” indicates “hypothesis could not be confirmed”, blank table field indicates that no test has been performed on the hypothesis. Clear evidence for the hypothesis is found for the intermediate phase. Reasons why the hypothesis could not be confirmed may include the measurements not being taken at the optimal time. The pattern of “+” and “–” suggests that it was too early in the early phase to detect an effect on blood glucose, and already too late in the late phase*.

a *Hypothesis could be confirmed by two independent experiments in one single paper*.

However, the papers that examined the late phase 3 to 7 days after reperfusion onset failed to confirm the hypothesis for the phase (Harada et al., 2009; Boujon et al., 2019). Two papers that examined the early phase could not confirm the hypothesis for this phase (Harada et al., 2009; Li et al., 2016), in contrast to Wang and coworkers who, as noted, could confirm it (Wang et al., 2013).

## Discussion

A total of 231 works was screened by title and abstract, and 89 were analyzed in full text. According to strict selection criteria defined in our PROSPERO pre-announcement and complying with PRISMA guidelines, 7 studies met all inclusion criteria. Of the 7 papers, all 5 that examined the intermediate phase 3 to 24 h after occlusion onset could confirm the hypothesis (Harada et al., 2009; Yamazaki et al., 2012, 2014; Wang et al., 2013, 2014) (Figure 4). For the late phase 3 to 7 days after reperfusion onset, the hypothesis could not be confirmed (Harada et al., 2009; Boujon et al., 2019). This evidence suggests that blood glucose elevation during and after cerebral artery occlusion is a temporary phenomenon. In all, our hypothesis could be fully confirmed for the period 3 to 24 h after the onset of a transient 2 h or permanent occlusion, so it holds that cerebral artery occlusion increases blood glucose concentration.

The 7 included studies represent a spectrum of diverse experiments which can be summarized as follows. All studies were published between 2009 and 2019, were performed on mice or rats, and the cerebral artery occlusion was either transient (ranging from 30 min to 2 h) or permanent (Harada et al., 2009; Yamazaki et al., 2012, 2014; Wang et al., 2013, 2014; Li et al., 2016; Boujon et al., 2019). Blood glucose was monitored with varying frequency over a period of about 2 h to 7 days. The papers that examined the intermediate phase reported that blood glucose concentrations 24 h after occlusion onset were 25 to 52 mg/dl higher than those of the control group (all p < 0.05). The papers that examined the late phase could no longer demonstrate the blood glucose increase seen in the intermediate phase (Harada et al., 2009; Boujon et al., 2019).

The mechanisms underlying stroke hyperglycemia are unknown, as some authors point out (Arnberg et al., 2015), while others hold overly complex beliefs (Dungan et al., 2009). Given that the Selfish-Brain theory makes accurate predictions in cases where the predictions of conventional theories failed (Sprengell et al., 2021), there is reason to believe that the neuroendocrine mechanisms as assigned by the Selfish-Brain theory provide a reliable explanation for the development of hyperglycemia in stroke. Typically, the Selfish-Brain theory covers topics such as psychosocial stress, depression, anorexia nervosa, obesity, and the development of atherosclerosis, myocardial infarction and stroke (Peters and McEwen, 2015). What is new here is that the theory addresses the consequences of stroke.

In terms of mechanisms, the Selfish-Brain theory refers to the principle of supply chains stating that when “push” fails, “pull” takes over. For the cerebral supply chain, this means that a reduction in the blood-push component (e.g. cerebral artery occlusion) is at least partially compensated by an enhanced brain-pull component. Brain-pull function is exerted by the sympathetic nervous system (SNS) and the hypothalamic-pituitary-adrenal (HPA) axis, which provide additional energy substrates for brain supply when needed. Among the studies found in our current systematic review, 2 studies measured blood concentrations of epinephrine, norepinephrine, and cortisol and showed that the concentrations of these stress hormones 24 h after occlusion onset were two times higher than those of the control group (Wang et al., 2013, 2014).

As mentioned above, several redundant brain-pull mechanisms are at work when the brain needs energy. Here we name three of them: The first brain-pull mechanism is that the SNS and HPA axis cause insulin suppression, which results in more glucose being delivered to the brain (Woods and Porte, 1974; Ahren, 2000). In fact, among the included studies was one that focused on insulin changes, showing that on day 1 after cerebral artery occlusion, an oral glucose load failed to increase plasma insulin concentrations, whereas in the sham-operated control group, the same load caused a 6-fold increase in plasma insulin concentrations (Harada et al., 2009). Thus, Harada and coworkers were able to confirm cerebral artery occlusion to induce CIS. CIS can be diagnosed on the basis of an inappropriately low insulin concentration at a given blood glucose concentration (Hitze et al., 2010). Importantly, CIS also occurs in many other critical situations, where brain energy homeostasis is challenged, including myocardial infarction (Taylor et al., 1969), psychosocial stress (Hitze et al., 2010), mental stress during anticipation of electric shock (Mason et al., 1968), acute hypoxia (Baum and Porte, 1969), deep hypothermia (Baum and Porte, 1971), caloric restriction (Peters et al., 2011) and sleep debt (Spiegel et al., 1999).

The second brain-pull mechanism is that SNS and HPA increase muscular proteolysis and hepatic gluconeogenesis, procuring the brain with even more glucose (Zhang et al., 2018). The third brain-pull mechanism is that SNS and HPA axis increase visceral lipolysis and hepatic ketogenesis, providing ketones as alternative brain substrate (Kubera et al., 2014). Indeed, it has been shown that brain ischemia via SNS activation induces the formation of ß-hydroxybutyrate (ketogenesis) in the liver and the consumption of ß-hydroxybutyrate in the brain (Koch et al., 2017). Ketones were also shown to exert beneficial effects on pathological and functional outcomes after experimental stroke (Gibson et al., 2012). Overall, brain-pull mechanisms cause the body stores to take in less energy and deliver more.

Replenishing the brain at the expense of body stores is what the supply chain model predicts will lead to weight loss (Figure 2B). One of the studies we included showed that compared to sham-operated controls, rats that underwent cerebral ischemia lost 9% of their body weight within 24 h (Wang et al., 2014). This is only a single experiment, and moreover, body weight was only a secondary endpoint. Nevertheless, these results match the prediction that weight loss in cerebral ischemia is caused by energy transfer from the periphery to the brain. Taken together, occlusion of the central cerebral arteries profoundly affects peripheral energy metabolism, with the strongest evidence for an increase in blood glucose concentration.

Hyperglycemia persists even after reperfusion, for which we here provide evidence of both cause and mechanism. In 3 of our included studies, hyperglycemia is still detectable 22 h after reperfusion (Harada et al., 2009; Yamazaki et al., 2012, 2014). While it is clear that impaired cerebral supply during occlusion leads to cerebral ATP deficiency (Wagner et al., 1992), the persistence of this deficiency state after reperfusion seems surprising at first glance. Regarding the cause of such a prolonged hyperglycemia there is evidence demonstrating that cerebral energy consumption increases from the onset of ischemia, particularly in the penumbra, but also in the core ischemic brain regions that undergo infarction (Arnberg et al., 2015). Cerebral energy consumption does not decrease until most ischemic brain regions have succumbed to infarction. As long as the penumbra exhibits increased energy consumption, cerebral ATP concentration as monitored in the VMH can recover only slowly. Thus, even after reperfusion, a prolonged hyperglycemic course seems plausible.

Regarding the mechanism accounting for increased energy consumption in undersupplied brain regions, the Selfish-Brain theory made the following predictions (Peters et al., 2004). ATP binds to low- and high-affinity ATP-sensitive-potassium channels localized on presynaptic GABAergic and postsynaptic glutamatergic neurons, respectively. This type of multi-site neuronal ensemble leads to biphasic responses when neuronal ATP concentrations fall. Mild ATP deficiency hyperpolarizes GABAergic neurons, disinhibits postsynaptic glutamatergic neurons, and thus increases glutamatergic activity and energy expenditure, whereas severe ATP deficiency hyperpolarizes glutamatergic neurons and thus suspends glutamatergic activity. Such a theory-predicted biphasic response to falling ATP levels could indeed be confirmed experimentally (Steinkamp et al., 2007). The clinical correlate of this biphasic course becomes evident when seizure susceptibility increases in moderate hypoglycemia due to facilitated glutamatergic activity, and coma develops in profound hypoglycemia due to ubiquitous silencing of glutamatergic activity (Arieff et al., 1974; Mobbs et al., 2001).

Post-stroke hyperglycemia has long been controversial, with either its deleterious-toxic, its beneficial neuroenergetic, or its Janus-faced dual aspect being held. The deleterious-toxic aspect was supported by the observation that higher blood glucose concentrations were associated with more severe ischemic strokes (Capes et al., 2001). On this basis, it was hypothesized that using insulin to normalize post-stroke hyperglycemia would improve infarct outcome. Several randomized controlled trials were conducted on this issue. Just as diabetologists and intensivists were disappointed with intensified insulin therapy because it caused more deaths in their type 2 diabetic and critically ill patients (Gerstein et al., 2008; The-NICE-SUGAR-Study-Investigators, 2009), so too were neurologists disappointed with intensified insulin treatment of post-stroke hyperglycemia. Specifically, a Cochrane review and the more recent SHINE trial showed that in ischemic stroke, intensified insulin therapy did not improve the outcomes of death, neurological deficit or dependency, but increased risk of symptomatic hypoglycemia (Bellolio et al., 2014; Johnston et al., 2019). While these findings from human studies challenged the deleterious-toxic aspect of post-stroke hyperglycemia, animal studies could indeed demonstrate glucotoxic mechanisms (Kruyt et al., 2010; Khan et al., 2016). This contradiction can be resolved with the Janus-faced dual aspect of post-stroke hyperglycemia, which allows for both deleterious-toxic and beneficial effects (Endres et al., 2008).

The beneficial neuroenergetic aspect of post-stoke hyperglycemia is also advocated (Arnberg et al., 2015). This position was supported by one of the larger randomized controlled trials on intensified insulin therapy, which showed that normalization of post-stroke hyperglycemia led to a 2.5-fold increase in infarct growth (Rosso et al., 2012). In line with this finding, Ginsberg and coworkers could show that rats rendered hyperglycemic by dextrose injection prior to infarct induction developed higher brain glucose concentrations and also smaller infarct volumes than normoglycemic controls (Ginsberg et al., 1987). At this point, a look at the evidence from diabetology and intensive medicine may provide further insight. Hypoglycemia and hypovolemia are among the situations critical for brain supply, in which CIS is essential for increasing blood glucose concentrations (Jarhult and Holst, 1978; Corrall and Frier, 1981). Presumably, neuroenergetic mechanisms such as CIS evolved under evolutionary pressure to survive periods of starvation or injury with blood loss, rather than to survive stroke. Nevertheless, hypoglycemia, hypovolemia, and ischemic stroke display the same neuroenergetic pattern: CIS with increase in blood glucose. This commonality supports the notion that infarct-related systemic hyperglycemia, despite its toxic aspect, is manifestation of an adaptive process that replenishes the energy-depleted brain.

Our systematic review has weaknesses and strengths. The weakness is that our search does not completely represent all relevant studies. However, no systematic review can claim to be complete unless the authors have viewed every paper in full text from all databases – which is not feasible. Authors of systematic reviews must therefore accept a certain degree of incompleteness due to the constraints imposed by their search algorithm. They need to find an optimal trade-off between sensitivity and specificity for their search. It became clear that our systematic review was not complete either when we had finished the data extraction of all papers. To our surprise, the work of Chen et al. (2016) surfaced. This work would have met all of our inclusion criteria, yet our search algorithm did not detect it. The reason for this was that the search term “ligation” did not appear in this paper, as Chen et al. referred to their previous work regarding their methods, nor was the term indexed for this paper in PubMed. We did not expect such a case when designing our search algorithm. We nevertheless adhered to the protocol and refrained from revising our search algorithm and performing a second search run, as such an approach risks biasing and undermining hypothesis testing.

One of the strengths of our systematic review is the neutrality of its search. We designed our search algorithm to the best of our knowledge and after that was set, had no influence on which studies were found. Even in the case of an incomplete search, this approach would provide us with an unbiased, well-defined set of experimental data against which we could perform hypothesis tests. Basically, compared to hand searching, systematic database searching is much less susceptible to researcher bias in paper selection. Now that we knew the results of Chen's study (Chen et al., 2016), a freedom of choice to either rerun a modified search or to stick with the existing search results would have provided us with an opportunity to influence hypothesis testing. To avoid such bias, we strictly followed our pre-registered PROSPERO protocol and stuck to our original search results. Nevertheless, and for the sake of completeness, we believe it is important to show whether Chen's results, had they been taken into account, would have affected the statement of our work. This was not the case (see Supplementary Material C). These considerations point out that when testing a scientific hypothesis in a systematic review it is the degree of completeness that matters, but even more so the neutrality of the search.

In conclusion, our systematic review confirms a major prediction of the Selfish-Brain theory, namely that cerebral artery occlusion elevates blood glucose concentrations. For the causes and effects involved in this blood glucose elevation, the theory makes further predictions such as increase in stress hormones epinephrine, norepinephrine and cortisol, suppression of insulin secretion, and acute body weight loss (Peters and McEwen, 2015), all of which could be fulfilled by findings from our included papers. This is the second major prediction of the Selfish-Brain theory that has been confirmed and that relates to a proximal bottleneck of the cerebral supply chain, the occlusion of the cerebral arteries. The first major prediction, which had also been confirmed, related to a distal supply bottleneck of the cerebral supply chain, caloric restriction (Sprengell et al., 2021). We did not only systematically search the literature databases, but also followed a systematic plan to capture the potential bottlenecks of the cerebral supply chain and predict their impact on cerebral and peripheral energy states. By passing this second test, centered on cerebral ischemia, the Selfish-Brain theory continues to demonstrate the accuracy of its predictions.

## Data Availability Statement

The raw data supporting the conclusions of this article will be made available by the authors, without undue reservation.

## Author Contributions

MS developed the search strategies that BK and AP approved. MS screened the article titles or abstracts against the inclusion and exclusion criteria. BK checked this step, and disagreements were resolved where necessary by consulting the third reviewer AP. MS and BK independently analyzed the full text, and disagreements were resolved where necessary by consultation with the third reviewer AP. MS extracted the data, which BK and AP independently checked. MS assessed the risk of bias, which BK and AP independently checked and approved. BK and AP wrote the manuscript. All authors have read and approved the submitted version.

## Conflict of Interest

The authors declare that the research was conducted in the absence of any commercial or financial relationships that could be construed as a potential conflict of interest.
