# Supplementary material for: Proximal Disruption of Brain Energy Supply Raises Systemic Blood Glucose: A Systematic Review
Source: Front Neurosci. 2021 Jun 24;15:685031. doi: 10.3389/fnins.2021.685031 (PMC8264130; doi:10.3389/fnins.2021.685031)
Supplement: Supplementary file 1 [file Data_Sheet_1.docx]

**SUPPLEMENTARY MATERIAL**

## **A. Search strategy MEDLINE via PubMED**

**#1 ((hypoxia-ischemia, Brain[MH] OR Infarction, Middle Cerebral Artery[MH] OR stroke[MH]) AND (ligation[MH] OR disease models, animals[MH])) OR “carotid ligation”[TI] OR “carotid ligature”[TI] OR (ligation[TI] AND “carotid artery”[TI]) OR (ligature[TI] AND “carotid artery”[TI]) OR (ligation[TI] AND ICA[TIAB]) OR (occlusion[TI] AND "carotid artery"[TI] AND model[TIAB]) OR (occlusion[TI] AND “Middle Cerebral Artery”[TI]) OR (occlusion[TI] AND MCA[TI]) OR MCAO[TI]OR (stroke[TI] AND “middle cerebral artery”[TI] AND model[TIAB]) OR (stroke[TI] AND MCA[TI] AND model[TIAB]) OR "focal cerebral ischemia"[TI] OR "transient forebrain ischemia"[TI] OR "permanent forebrain ischemia"[TI] OR ("cerebral ischemia"[TI] AND model[TIAB]) OR “experimental stroke”[TI]OR (stroke[TI] AND model[TI]) OR “two vessel occlusion”[TI] OR "2 vessel occlusion"[TI] OR 2VO[TI] OR (thromboembolic[TI] AND stroke[TI] AND model[TIAB]) OR “Intraluminal suture”[TI]**

**#2 Blood Glucose[MH] OR “blood glucose”[TIAB]OR "blood sugar"[TIAB]**

**#3 Animal Experimentation[MH] OR Animals[MH] OR Random Allocation[MH] OR randomized controlled trial[PT] OR clinical trial, veterinary[PT] OR randomization[ALL] OR experiment[ALL] OR intervention*[TIAB] OR groups[TIAB] OR randomly[TIAB] OR randomized[TIAB] OR trial[TIAB] OR “animal study”[TIAB] OR “control group”[TIAB]**

**#4 #1 AND #2 AND #3**NOT review[ALL] NOT patients[TI]NOT women[TIAB] NOT men[TIAB]**NOT cohort[TIAB]**

## **B. Search Strategy Biosis Previews via Biosis Citation Index**

#1 TI=(((“ischemia” OR “infarction” OR “stroke”) AND (“ligation” OR “model”)) OR **“carotid ligation” OR “carotid ligature” OR (“ligation” AND “carotid artery”) OR (“ligature” AND “carotid artery”**) **OR (“ligation” AND “ICA”) OR (“occlusion” AND "carotid artery" AND “model”) OR (“occlusion” AND “Middle Cerebral Artery”) OR (“occlusion” AND “MCA”) OR “MCAO” OR (“stroke” AND “middle cerebral artery” AND “model”) OR (“stroke” AND “MCA” AND “model”) OR "focal cerebral ischemia" OR "transient forebrain ischemia" OR "permanent forebrain ischemia" OR ("cerebral ischemia" AND “model”) OR “experimental stroke” OR “two vessel occlusion” OR "2 vessel occlusion" OR “2VO” OR (“thromboembolic” AND “stroke” AND “model”) OR “Intraluminal suture”)**

#2 TS=(**“blood glucose” OR "blood sugar"**)

#3 TS=(**“Animal Experimentation” OR “Animals” OR “Random Allocation” OR “randomized controlled trial” OR “randomization” OR “experiment” OR “intervention$” OR “groups” OR “randomly” OR “randomized” OR “trial” OR “animal study” OR “control group”**)

#4 #1 AND #2 AND #3 NOT TS=(“review” OR “patients”OR “women” OR “men”**OR “cohort”**)

**C. Supplemental**

Chen and colleagues showed that blood glucose concentrations 2 hours after the onset of permanent cerebral occlusion were higher in the intervention group than in the sham-operated group (baseline 80±25 mg/dl vs 55±10 mg/dl, n.s.; 2h-values 270±85 mg/dl vs 75±15 mg/dl, P<0.05; mean±SD values taken from the graph) (Chen et al., 2016). Thus, Chen's results too could confirm our hypothesis that cerebral artery occlusion increases blood glucose concentration.

Chen, W.Y., Mao, F.C., Liu, C.H., Kuan, Y.H., Lai, N.W., Wu, C.C., Chen, C.J. (2016). Chromium supplementation improved post-stroke brain infarction and hyperglycemia. Metab Brain Dis 31, 289-297.
